# Synchronous cervical necrotizing fasciitis and pharyngocutaneous fistula: A case report

**DOI:** 10.1016/j.ijscr.2022.106988

**Published:** 2022-03-29

**Authors:** Saleh Al-wageeh, Faisal Ahmed, Qasem Alyhari, Fawaz Mohammed

**Affiliations:** a Department of General Surgery, School of Medicine, Ibb University of Medical Science, Ibb, Yemen; b Urology Research Center, Al-Thora General Hospital, Department of Urology, School of Medicine, Ibb University of Medical Science, Ibb, Yemen; c Department of Orthopedy, School of Medicine, Ibb University of Medical Science, Ibb, Yemen

**Keywords:** Case report, Fasciitis, Pharyngocutaneous fistula, Infection, Neck, Necrotizing

## Abstract

**Introduction and importance:**

Necrotizing fasciitis is a fulminant infection that spreads along the fascial planes. It is a rare entity with potentially fatal outcomes. The head and neck involvement is infrequent, with primary source either odontogenic or pharyngeal infection by single or mixed bacterial flora. To our knowledge, synchronous cervical necrotizing fasciitis (CNF) and pharyngocutaneous fistula is rarely reported in pieces of literature.

**Case presentation:**

We present a 38-years-old female patient who presented with CNF and pharyngocutaneous fistula. Diabetes mellitus was accidentally discovered during the investigation. The patient was successfully treated with broad-spectrum antibiotics, serial surgical debridement sessions, wound irrigation, and multiple muscular and myocutaneous skin flaps.

**Clinical discussion:**

Rapid diagnosis, radical surgical debridement of all necrotic tissue, intravenous broad-spectrum antibiotics, and close monitoring of patients with CNF are crucial to avoid critical complications and better patient survival. Due to the poor healing process in the neck area, the pharyngocutaneous fistula should be repaired with good musculocutaneous flaps such as the pectoralis major myocutaneous flap. Meticulous suturing of the flap to the mucosa, reinforcement of the repair with muscle, and suturing of the skin without tension are essential to obtaining a successful outcome.

**Conclusion:**

Synchronous CNF and pharyngocutaneous fistula are rare events. Initial diagnosis and serial surgical debridement, along with aggressive broad-spectrum antibiotics and adequate resuscitation with great attention to the poor healing process in the diabetic patients' neck area, are critical for a beneficial result. In our case, the reconstruction was performed successfully using multiple muscular and skin flaps.

## Highlights

• Cervical necrotizing fasciitis (CNF) is a fetal infection with a high mortality rate even under ideal medical conditions.
• Synchronous CNF and pharyngocutaneous fistula are rare occurrence.
• In CNF with pharyngocutaneous fistula, initial diagnosis, surgical debridement, broad-spectrum antibiotics and resuscitation with attention to poor healing process, are critical for a beneficial result.

## 1 Introduction

Necrotizing fasciitis (NF) is a virulent infection that expands along the fascial planes, causing vascular thrombosis, necrosis of the skin, and other surrounding tissues [1], [2]. Although it is a rare entity, it is associated with more significant systemic toxicity and increased morbidity and mortality [1]. Adult occurrence of NF has been reported to be 0.4 cases per 100,000 people [1]. Lower extremities (28%) were the most frequently infected site, followed by upper extremities (27%), genital area (21%), and trunk (21%) [3].

Cervical necrotizing fasciitis (CNF) is much relatively rare (5%) and has only a few cases reported in the forties [4], [5]. It was first described as “hospital gangrene” during the American civil war until 1952 when Wilson used the term CNF to describe the etiology and debated mixed flora as a potential cause [6]. Although the origin of infection is usually odontogenic or pharyngeal, with group A-beta hemolytic Streptococcus, it usually involves mixed flora [1], [5].

Depending on the wound culture, the CNF is classified as Type 1; polymicrobial, caused by a mixed aerobic and anaerobic infection. Type 2; monomicrobial infection is caused by group A Streptococcus and, in rare cases, methicillin-resistant *Staphylococcus aureus*. These CNF infections are complicated by toxic shock syndrome in up to 50% of cases, with positive blood cultures in approximately 60% of cases. Type 3; gas gangrene infections which are most commonly caused by the anaerobe *Clostridium perfringens* [7].

Early disease diagnosis, radical surgery, and broad-spectrum antibiotic therapy are critical in improving prognosis. While, delayed treatment or misdiagnosis can have disastrous consequences, with a mortality rate ranging from 15% to 40% [5]. We present a rare case of synchronous CNF and pharyngocutaneous fistula in a 38-years-old female; the disease presentation, primary management, and outcome are discussed. A senior general surgeon performed the surgery in a teaching university hospital (Al-Thora General Hospital, Ibb University, Ibb, Yemen). This case report has been reported in line with the SCARE Criteria [8].

## 2 Presentation of the case

### 2.1 Patient information

A 38-years-old female patient was referred to our surgery department at Al-Thora General Hospital, Ibb, Yemen, in November 2021, with a chief complaint of offensive gangrenous neck ulceration started seven days ago. This condition started as neck pain and swelling with mild sore throat and difficulty in speech with some choking and dysphagia. The swelling increases progressively on the right side and becomes diffuse, associated with mild redness and low-grade fever.

The patient begins to do soothing (ichthammol) ointment to prepare the area for spontaneous drainage. However, the condition became more complicated, the skin became dark, and the redness spread more and more, reaching the upper chest, and spontaneous rupture occurred in the right side of the neck with offensive discharge associated with water and saliva leakage through the defect.

Regarding her medical history, the patient gave a history of spontaneous weakness and easy extraction of her teeth attributed to a low calcium level two years ago, and had a plastic denture for her lower teeth seven months ago.

### 2.2 Clinical findings

**Fig. 1:**
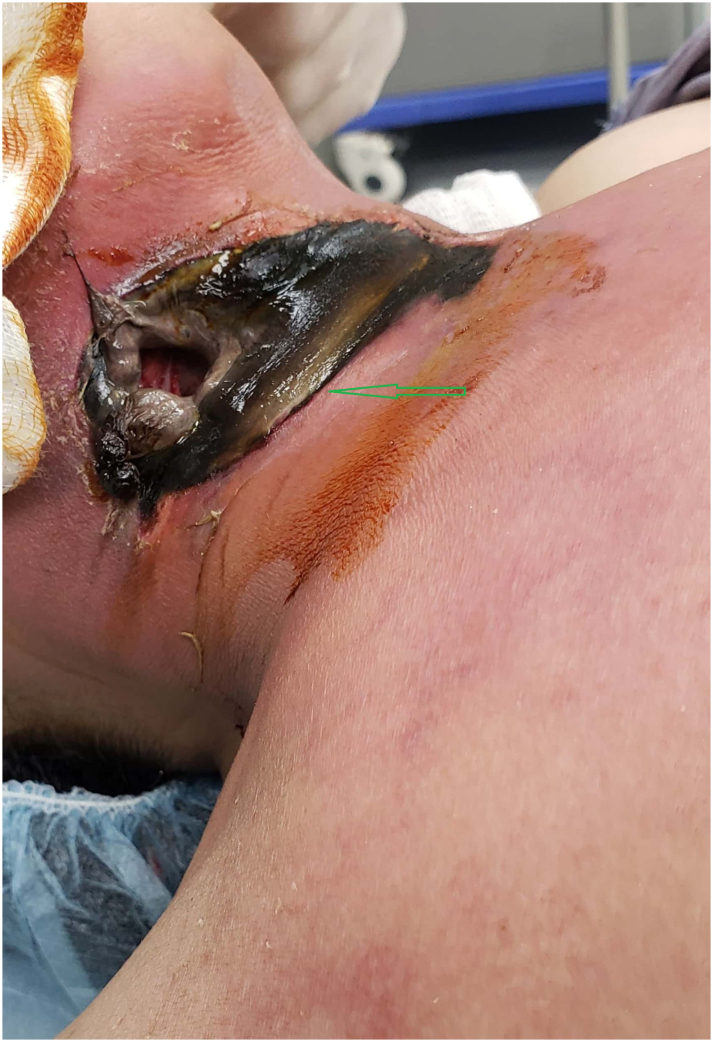
Showing cervical necrotizing fasciitis (arrow).

On physical examination, the patient was conscious, oriented, looked ill, but not pale or cyanotic. The pulse rate was 90 beats/min, the respiratory rate was 20/min, the blood pressure was 130/60 mm/Hg, and the oral temperature was 38 °C. The oral cavity was normal. Cervical area examinations revealed gangrenous skin and soft tissue on the anterior and right side of the neck with an opening in the right upper neck in which air and saliva pass through it (Fig. 1). There is redness and shininess in all neck and the upper chest wall with edematous skin and palpable crepitus.

### 2.3 Diagnostic assessment

White blood cell count: 14.29 × 10^3^/ml with predominant neutrophilia (74%), hemoglobin: 14.5 g/dl, platelets count: 351 g/dl, serum creatinine: 0.8 mg/dl, glycated hemoglobin (HBA1C): 10.9, serum potassium: 3.2 mEq/l and serum sodium: 134 mEq/l. During the investigation, high blood sugar was accidentally discovered (276 mg/dl). The patient was prepared emergently for surgical operation without ultrasonography or computed tomography (CT) scan investigations.

### 2.4 Therapeutic interventions

Firstly, the patient received hydration with 1000 cm^3^ normal silane, insulin, and empiric broad-spectrum antibiotics [piperacillin/tazobactam (4 g/0.5 g) intravenously every 8 h and metronidazole 500 mg intravenously every 8 h]. After consent was obtained, she was transferred to the surgery room.

**Fig. 2:**
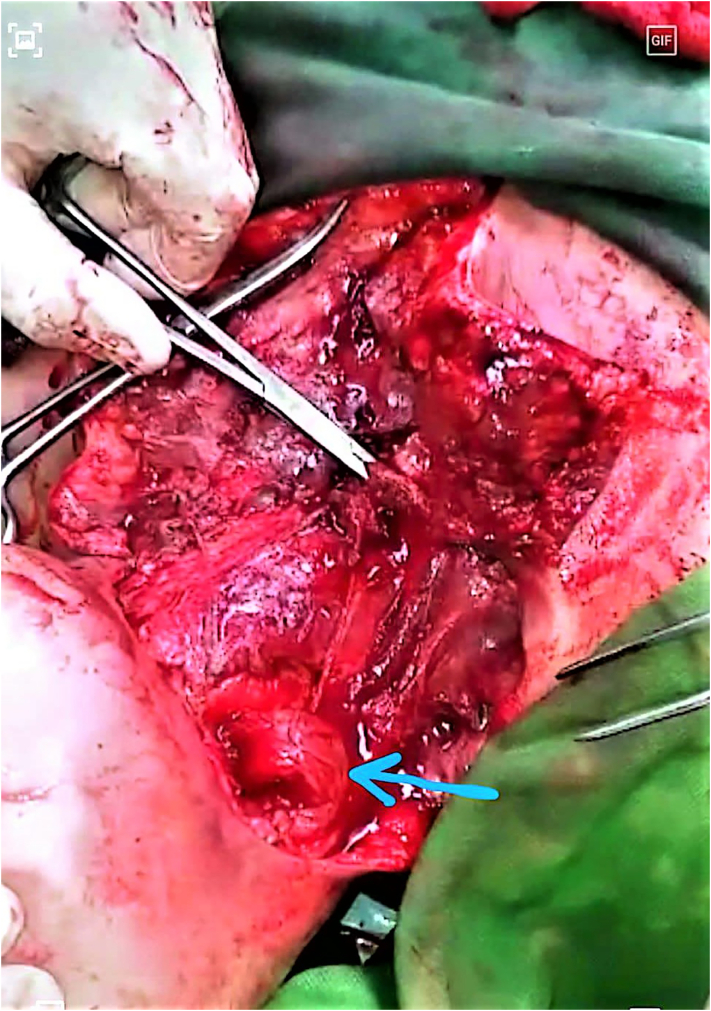
Intraoperative photo showing the closing pharyngocutaneous fistula by the right-side sternocleidomastoid muscle flap (arrow).

Under general anesthesia with supine and extended neck position and after patient preparation and draping, necrotic tissue including skin, fascia, and platysma were radically debrided with extension of the neck incision to the upper chest wall. The pus was taken and sent for culture. Meticulous irrigation with normal saline and diluted betadine was performed. The pharyngocutaneous fistula was closed by the right-side sternocleidomastoid muscle flap (Fig. 2). The nasogastric tube (NG) was inserted. Finally, the wound was left open for serial wound debridement and dressing.

We admitted the patient to the surgical intensive care unit (ICU) for monitoring and diabetic control. NG tube feeding was started the next day and continued for two weeks with good compliance. Bacterial growth revealed Gram-positive *Staphylococcus aureus*, which changed antibiotic therapy based on culture sensitivity (ciprofloxacin 400 mg intravenous infusion every 12 h).

After two weeks of serial wound dressing and debridement, the wound was clean; however, there was a salivary leak medial to the muscular flab. The patient was transferred to the operating room again and under general anesthesia, we performed a myocutenous pectoralis flap to cover the skin defect and support the closure of the fistula. The patient was transferred to the surgical ICU for three days and the patient resumed oral feeding without any leakage. Then, the patient was shifted to the general surgery department for regular dressing and debridement, which continued for two weeks.

Two weeks postoperative, the patient was admitted for exposed raw area coverage. During intubation, the anesthesiologist had some difficulty. We performed a local rotational flap to cover the exposed part of the neck. Finally, the patient was discharged home on the third postoperative day with regular dressing, insulin treatment, and a visit to the outpatient clinic. A senior general surgeon performed all surgical procedures.

### 2.5 Follow-up and outcome

**Fig. 3:**
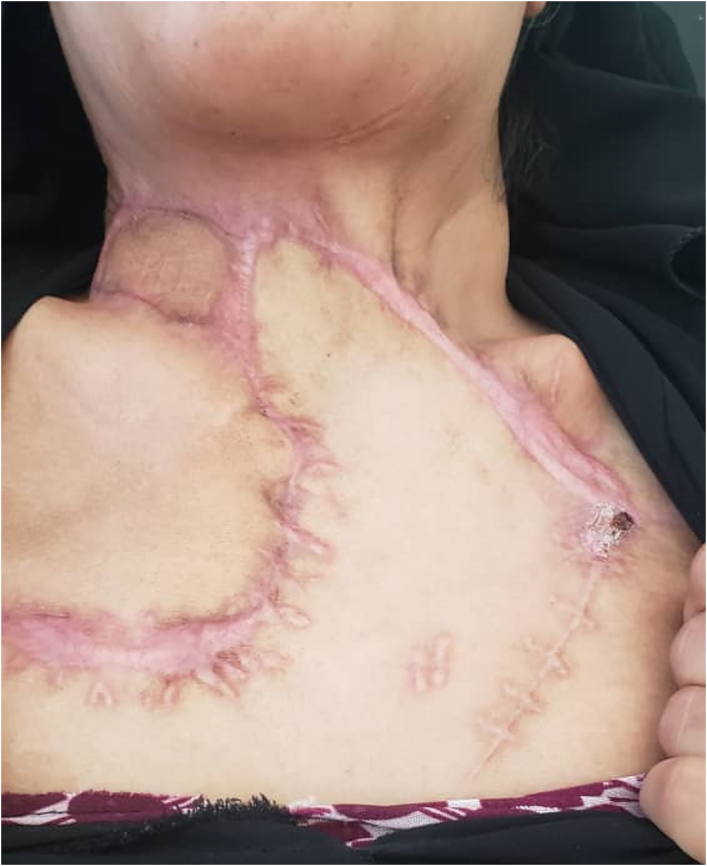
The 4th-week postoperative photo showing complete healing with good appearance of skin.

At two weeks postoperative follow-up, the skin had a good appearance, and the patient was satisfied with the final results (Fig. 3). The patient was advised to regular follow-up with an endocrinologist for blood sugar control and a general surgeon for follow-up.

## 3 Discussion

We report a rare case of CNF with pharyngocutaneous fistula, which was successfully treated with antibiotic therapy, surgical debridement, and multiple flaps. NF is a fetal soft tissue infection with up to 50% mortality rates even under optimal medical conditions [9]. CNF is predominantly odontogenic or pharyngeal in origin and can occur in patients of any age, without preference for gender or ethnicity [10]. The most common risk factor is a peritonsillar abscess, followed by diabetes mellitus [1], [11]. Chronic renal failure, cancer, ankylosing spondylitis, and intravenous drug abuse are also risk factors [1], [12]. Our patient accidentally discovered diabetes mellitus and did not have another predisposing factor.

CNF can present with localized and systemic clinical manifestations. Local CNF manifestations are similar to NF at other sites and include skin blistering, erythema, swelling, crepitus, odorous, watery discharge from wounds, and a dramatic worsening of the patient's condition. Multisystem organ failure due to widespread septic shock caused by bacteremia and exotoxin release is the systemic CNF manifestations [13], [14].

External jugular vein thrombosis, arterial erosion, mandibular necrosis, formation of lung and mediastinal abscesses, and chest wall necrosis are all complications of CNF [13]. Although CNF is a complication of various primary head and neck infections, Lee et al. reported that primary infections were not discovered in 73% of their cases [15]. Our patient developed a pharyngocutaneous fistula, which is a rare complication. Pharyngo-cutaneous fistula as a complication of CNF was reported by Bahu et al. and Koizumi et al. [16], [17].

The culture result in our patient showed a gram-positive *Staphylococcus aureus*. There are different bacteria isolated from these infections. Horvath et al. reported aerobic in 9 (53%) cultures, including 7 (41%) Gram-positive *Staphylococcus aureus* and anaerobic in 6 (35%) cultures [18]. The various isolated bacteria could be related to geographic locations and epidemiology [13].

Various radiologic imaging modalities can aid in the diagnosis by accurately identifying the affected areas of the neck and demonstrating disease behaviors [13]. CT scan, US, and Magnetic resonance imaging (MRI) are preferred radiologic modalities [13]. However, we did not perform any radiologic investigations because our patient was toxic, and there was no additional time to investigate. Additionally, the patient was of low socioeconomic status and did not have any money to perform the US or CT scan. For that, we transferred the patient for emergency surgical exploration depending on our clinical findings.

Early detection and aggressive treatment of CNF are critical for successful management, good results, and avoiding the high risk of mortality [1], [19].

Treatment should include immediate and radical surgical debridement of all necrotic tissue to control the infectious process and toxin production [1]. If the necrotic soft tissue infection is not sufficiently debrided, the patient may experience septic shock and can only be saved if the patient is immediately returned to the surgery room for more substantial operative debridement [19]. We performed a radical debridement of affected necrotizing tissues and muscles. However, several operations were performed to repair the fistula, supplementary debridement, and flap coverage.

Antibiotic therapy with complete coverage of the most common organisms should be started immediately, followed by narrowing intravenous antibiotics once culture sensitivities are obtained [16]. Adequate resuscitation of intravenous fluid and close monitoring should also be applied. Similar to our procedure, Bahu et al. and Al-Ali et al. advocated repeat wound exploration and debridement for a better outcome [9], [16].

The regimens of broad-spectrum empiric antibiotics differ by country [16]. The first line of defense should include the most commonly involved microorganisms, such as group A Streptococcus and anaerobes, and is now expanded to include Gram-negative and Staphylococcus. Typically, the regimen begins with a triple therapy of a beta-lactamic acid, an aminoglycoside, and clindamycin or metronidazole. The most commonly used medications in the reported series were amoxicillin/clavulanate, penicillin, imipenem, aminoglycosides, clindamycin, and metronidazole [4], [14]. The empiric antibiotic therapy should be changed based on the culture sensitivity results [16].

In our case, the empiric antibiotics were piperacillin/tazobactam and metronidazole and were changed to ciprofloxacin after culture sensitivity.

Regarding pharyngocutaneous fistula and its management, Koizumi et al. reported a case of CNF with pharyngocutaneous fistula in a 74-year-old woman and the fistula repaired by pectoralis major myocutaneous flap [17]. Due to the rich muscle volume and consistent regional blood flow, the authors stated that a major myocutaneous flap is suitable for reconstructing a significant pharyngeal and cervical defect with a poor situation for the healing process [17]. In our case, we performed a sternocleidomastoid muscular flap, a myocutenous pectoralis flap, and a fasciocutaneous flap. We begin to close the pharyngocutaneous fistula with the sternocleidomastoid muscular flap during the first CNF debridement. Then after serial debridement and resolving of the edematous tissue. A small opening has remained near to the flap. We performed a myocutenous pectoralis flap to batter the previous muscular flap and close the skin defect. We did not use the deltopectoral flap due to the involvement of all chest walls with infection, making it an unreliable flap.

Furthermore, the causes of the next fasciocutaneous flap in our case were loss of the partial part of the skin due to vascular thrombosis and the severity of infection [20]. A high success rate for fistula closure can be achieved with meticulous suturing of the flap to the mucosa and strengthening the repair with muscle [20]. Furthermore, the scarred neck skin must be removed liberally and no attempt should be made to advance the surrounding skin to reduce or close the defect [20].

In patients with CNF, maintaining a high suspicion regarding damage to shrouding organs such as our patient, who presented with pharyngocutaneous fistula, is essential to minimize the complication rate and obtain the optimal outcome.

## 4 Conclusions

CNF is a rare but fatal infection. Maintaining a high level of suspicion is critical. Early diagnosis of CNF, resuscitation and radical surgical debridement of necrotic tissue are critical components for an effective outcome. Early repair of the pharyngocutaneous fistula with sternocleidomastoid muscular flap and transposition of the myocutenous pectoralis flap was influential in our patient.

## Patient perspective

The patient was happy with the successful outcome of the surgery.

## Informed consent

Written informed consent was obtained from the patient for publication of this case report and accompanying images. A copy of the written consent is available for review by the Editor-in-Chief of this journal on request.

## Provenance and peer review

Not commissioned, externally peer-reviewed.

## Ethical approval

Not required.

## Funding

This research did not receive any specific grant from funding agencies in the public, commercial, or not-for-profit sectors.

## Guarantor

Faisal Ahmed is the guarantor of the work and accepts full responsibility.

## Research registration number

N/a.

## CRediT authorship contribution statement

Study concept and design: Saleh Al-wageeh and Faisal Ahmed
Data collection: Qasem Alyhari, Fawaz Mohammed
Writing of paper: Saleh Al-wageeh and Faisal Ahmed
Critical revision for intellectual content: Saleh Al-wageeh and Faisal Ahmed.

## Declaration of competing interest

None of the authors have any conflict of interest to declare.
